# Quantitative transcriptomic and metabolic analyses reveal the roles of RpoS and Crp in the acid resistance system 1 in *Escherichia coli*

**DOI:** 10.1128/spectrum.02063-25

**Published:** 2026-06-15

**Authors:** Wenbin Zhang, Liang Zhang, Jinxin Xu, Qinghua Huang, Ling Wang, Huohua Liu, Xin Chen, Zhonghai Sun, Qirui Zhou, Yujian Wu, Tianli Yu, Haihong Wang, Wei Lei, Yirong Sun

**Affiliations:** 1 Guangdong Provincial Key Laboratory of Stem Cell and Regenerative Medicine, Guangzhou institutes of Biomedicine and Health, Chinese Academy of Sciences 53042 , Guangzhou, China; 2 Guangdong Provincial Key Laboratory of Protein Function and Regulation in Agricultural Organisms, College of Life Sciences, South China Agricultural University 12526, Guangzhou, China; 3 Research Center of Translational Medicine, Jinan Central Hospital Affiliated to Shandong First Medical University 518873, Jinan, Shandong, China; 4 The Department of Dermatology, the Second Affiliated Hospital of Guangzhou Medical University 26468, Guangzhou, China; 5 Guangdong Key Laboratory of IoT Information Technology, School of Automation, Guangdong University of Technology 47870, Guangzhou, China; 6 Department of Pharmaceutical and Graduate Life Sciences, College of Health Professions, Nursing and Pharmacy, Manchester University 6061, Fort Wayne, Indiana, USA; Zhongxiong Lai, Fujian Agriculture and Forestry University, Fuzhou City, Fujian, China

**Keywords:** acid resistance, RpoS, Crp, transcriptomics, metabolic adaptation, *Escherichia coli*

## Abstract

Foodborne pathogens exploit acid resistance (AR) mechanisms to survive harsh environments, such as the stomach, increasing contamination risk. While the sigma factor RpoS and cyclic AMP receptor protein (Crp) are known mediators of AR, their systemic regulatory networks remain unclear. Here, we integrated transcriptomics and metabolomics to dissect RpoS- and Crp-dependent responses in *E. coli* under simulated gastrointestinal neutral (pH 7.5) and acidic (pH 5.5) conditions. We identified 78 coregulated genes, with RpoS dominating energy/amino acid metabolism and Crp primarily modulating amino acid pathways. Both regulators upregulate membrane components (fatty acids, chaperones, transporters) essential for AR, with transporters NarU (RpoS-dependent) and AmtB (Crp-dependent) conferring exceptional survival at pH 2.5. Surprisingly, Crp activates flagellar genes, whereas RpoS suppresses them during acid stress. Our work provides a systems-level understanding of AR adaptation, offering translational potential for antimicrobial development and bioremediation strain engineering.

**IMPORTANCE:**

*Escherichia coli* and other gut bacteria colonize or infect the extremely acidic environment of the gastrointestinal tract through the acid resistance mechanism. Therefore, it is necessary to investigate the acid resistance mechanism of intestinal bacteria. Although RpoS and Crp have long been found to regulate the acid resistance of *E. coli*, there is a lack of systematic research to analyze its regulatory network under the acidic environment. Through an integrated approach combining genomic analysis, we obtained robust research findings. These results establish a novel theoretical framework for understanding microbial adaptation to acidic environments while offering potential applications for developing new antibiotic targets against intestinal pathogens and engineering industrial bacterial strains with enhanced acid tolerance capabilities.

## INTRODUCTION

Foodborne pathogens pose a significant global health burden, causing approximately 600 million illnesses annually (1). The mammalian stomach’s extreme acidity (pH ~2.0) serves as a natural antimicrobial barrier (2, 3), yet enteric bacteria, such as *Escherichia coli* and *Salmonella enterica* , evade this defense via acid resistance (AR) and acid tolerance response (ATR) (4–6). AR and ATR are induced by moderate acid stress (pH 4.5–5.8), with ATR enabling bacterial growth at acidic conditions, while AR protects against subsequent extreme acidity (pH 2.0–3.0) (6, 7). Although numerous genes and metabolic adaptations contribute to AR/ATR, their regulatory networks remain poorly defined (6–9).

The human stomach’s pH fluctuates from ~5.0 during meals to ~2.0 postprandially, allowing foodborne bacteria to undergo AR/ATR adaptation (10). This provides an opportunity for bacteria to obtain AR throughout the entire adaptation process under weakly acidic pH or ATR (11). Notably, *E. coli* O157 maintains AR or ATR in meat, possibly due to pre-adaptation in the weakly acidic rumen of cattle (11–14). Understanding how *E. coli* transitions from moderate to extreme acid survival is critical for elucidating gut colonization strategies.

Multiple genes and molecules have been reported to contribute to the AR of intestinal bacteria, which allows commensal and pathogenic enteric bacteria to fight acidic stress (2, 12, 15). Four AR systems (AR1–AR4) have been identified in enteric bacteria (2). While AR2–AR4 rely on amino acid decarboxylation (glutamate, arginine, and lysine) (2), AR1 induced in stationary or logarithmic phase under moderate acidity requires the sigma factor RpoS and the cAMP receptor protein (Crp) (2, 16–19). Despite their known roles, the global regulatory mechanisms of RpoS and Crp in AR1 remain unclear.

In this study, we systematically dissect RpoS- and Crp-dependent gene regulation and metabolic remodeling in *E. coli* under varying pH conditions. Through comparative transcriptomics of deletion mutants across pH conditions, AR functional assays, and metabolic profiling, we identified key genes and pathways involved in acid adaptation. Our integrated analysis reveals novel insights into the coordinated regulation of membrane transporters, metabolic pathways, and flagellar systems by these global regulators. These findings advance our understanding of bacterial acid survival strategies and may inform new approaches for controlling foodborne pathogens or engineering acid-resistant industrial strains.

## RESULTS

### Crp and RpoS are essential for AR in *E. coli*, with distinct regulatory roles across growth phases and pH conditions

**Fig 1:**
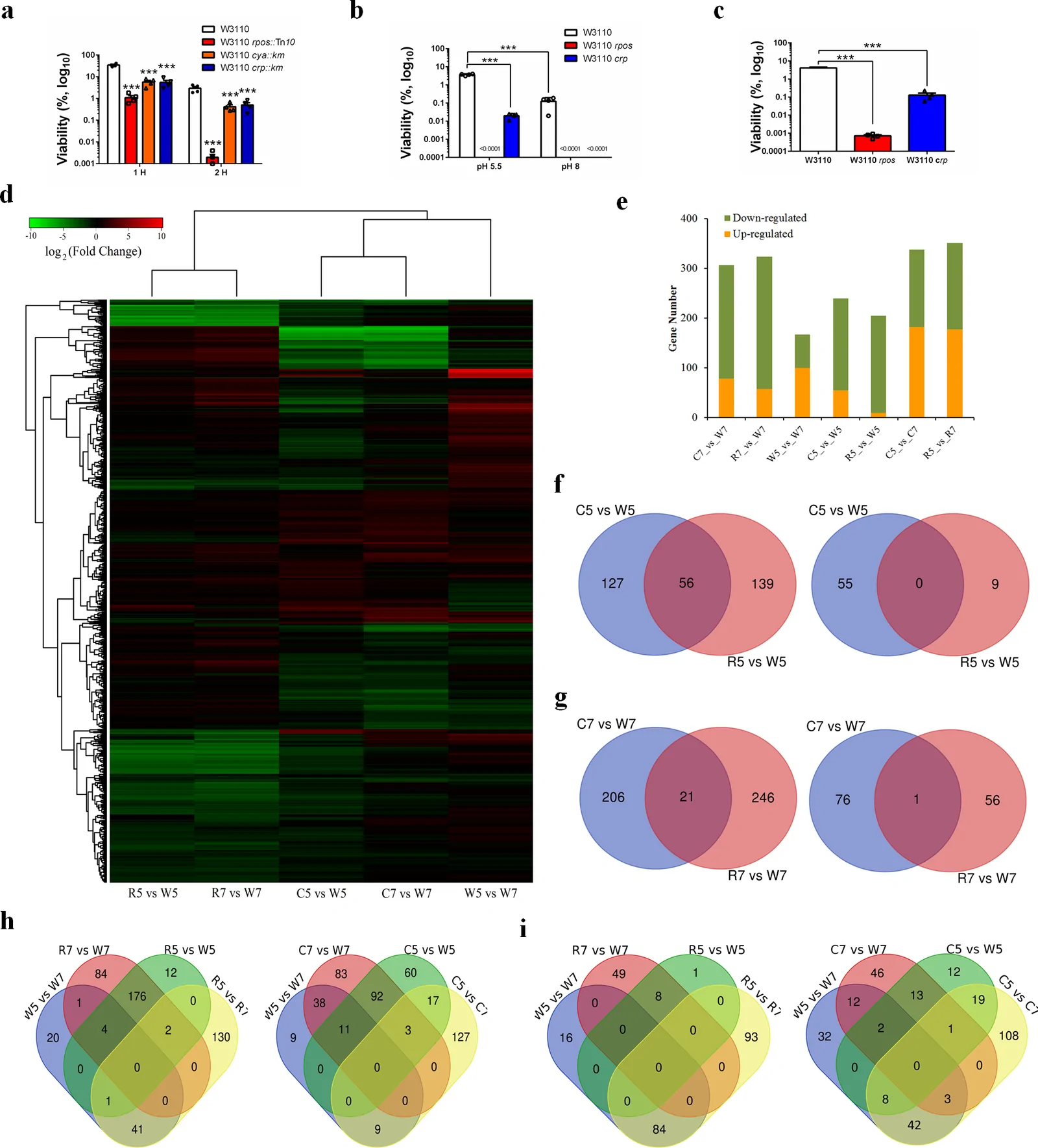
RpoS and Crp are important for AR during the logarithmic and stable phases. (**a**) The survival rates of wild-type *E. coli* W3110 and W3110 *rpoS*::Tn*10*, W3110 *cya::km*, and W3110 *crp::km* decreased under extremely acidic pH conditions after adaptation at pH 5.5 for 3 h during the logarithmic growth phase. (**b**) ARs of W3110, W3110Δ*rpoS*, and W3110Δ*crp* at stationary phase for challenge at pH 2.5 for 2 h. (**c**) Survival rates of W3110, W3110Δ*rpoS*, and W3110Δ*crp* strains after challenge at pH 2.5 for 2 h and acid adaptation at pH 5.5 at the logarithmic phase for 3 h. (**d**) Heatmap of fold changes in the wild type and mutants at different pH values. (**e**) Number of up- and downregulated genes in the wild type and mutants at different pH values. (**f**) Venn diagram of W3110Δ*crp-* and W3110Δ*rpoS*-downregulated genes (left) and upregulated genes (right) at pH 5.5; (**g**) Venn diagram of W3110Δ*crp-* and W3110Δ*rpoS*-downregulated genes (left) and upregulated genes (right) at pH 7.5; (**h**) Venn diagram of genes downregulated in W3110 at pH 5.5 relative to pH 7.5, compared with downregulated genes in the wild-type W3110Δ*crp* (right) and W3110Δ*rpoS* (left) at different pH values. (**i**) Venn diagram of genes upregulated in W3110 at pH 5.5 relative to pH 7.5 compared with upregulated genes in the wild-type W3110Δ*crp* (right) and W3110Δ*rpoS* (left) at different pH values. W5, C5, and R5 indicate that W3110, W3110Δ*rpoS*, and W3110Δ*crp*, respectively, were grown at pH 5.5 in EG media. W7, C7, and R7 indicate that W3110, W3110Δ*rpoS*, and W3110Δ*crp*, respectively, were grown at pH 7.5 in EG media. Bars represent mean ± standard deviation (*n* = 3*–*5). One-way analysis of variance (***, *P* < 0.001).

The RpoS and Crp genes have been reported to play key roles in stationary phase cells and log phase ARs in *E. coli* (20, 21). We found that mutation of *crp* or *rpoS* severely impaired survival during 2-h exposure to pH 2.5, reducing viability by 10-fold and 100-fold, respectively, compared with wild-type strain W3110 (WT). The survival rate of WT after 1 and 2 h of extreme acid treatment were 34.50% and 2.98% (Fig. 1a). Complete ORF deletions confirmed by Red recombination (Fig. S1a and b) eliminated polar effects; Δ*crp* abolished β-D-galactosidase activity (22) (Fig. S1c and d) and both mutants exhibited delayed growth at pH 7.0 in EG medium (Fig. S1e). Pre-adaptation to pH 5.5 failed to rescue survival defects in either mutant. Δ*crp* and Δ*rpoS* showed significant viability loss in both log phase (Fig. 1b) and stationary phase (Fig. 1c), with Δ*rpoS* consistently exhibiting stronger phenotypes, that is, a lower survival rate.

RNA-seq profiling of pH 5.5-adapted cells revealed 167 differentially expressed genes (67 downregulated, 100 upregulated) in WT cultured at pH 5.5 (W5) versus WT cultured at pH 7.5 (W7). Δ*crp* and Δ*rpoS* deletions further affected transcripts under different pH conditions compared with the wild type (Fig. 1d and e; Table S1). Core acid-response pathways (56 genes) were downregulated in both mutants (Fig. 1f; Table S2), while pH 7.5-specific regulation involved 22 genes (21 downregulated, 1 upregulated; Fig. 1g).

Gene-specific analyses highlighted Δ*rpoS* suppressed *aldB*, *aroM*, *ydcT*, and *ydcS* expression across pH conditions and uniquely downregulated *bsmA* at pH 5.5 (Fig. 1h left; Table S3), whereas Δ*crp* constitutively repressed 11 genes and pH-dependently silenced 38 additional genes at pH 7.5 (Fig. 1h right; Table S3). qPCR results further confirmed these findings, which showed that Δ*rpoS* suppressed the expression of *aldB*, *aroM*, *ydcS*, and *ydcT* at both pH 7.5 and 5.5 (Fig. S1f and g), and downregulated *bsmA* only at pH 5.5 (Fig. S1h). Adaptation to pH 5.5 upregulated 100 genes in WT, a response unaffected by Δ*rpoS* but partially disrupted in Δ*crp*, which induced distinct activation of 17 genes at pH 7.5 and 10 genes at pH 5.5, including constitutively expressed *gspE* and *yjcB* (Fig. 1i; Table S3). As further confirmed by the qPCR results, Δ*crp* upregulated *gspE* and *yjcB* at both pH 7.5 and 5.5 (Fig. S1i).

These data indicate that RpoS and CRP involved in AR are complex and may play a role by regulating the overall metabolic processes and gene expression of *E. coli*. In the following sections, we will further analyze how Crp and RpoS regulate metabolic pathways related to AR.

### Crp and RpoS orchestrate a comprehensive amino acid metabolic network for AR

**Fig 2:**
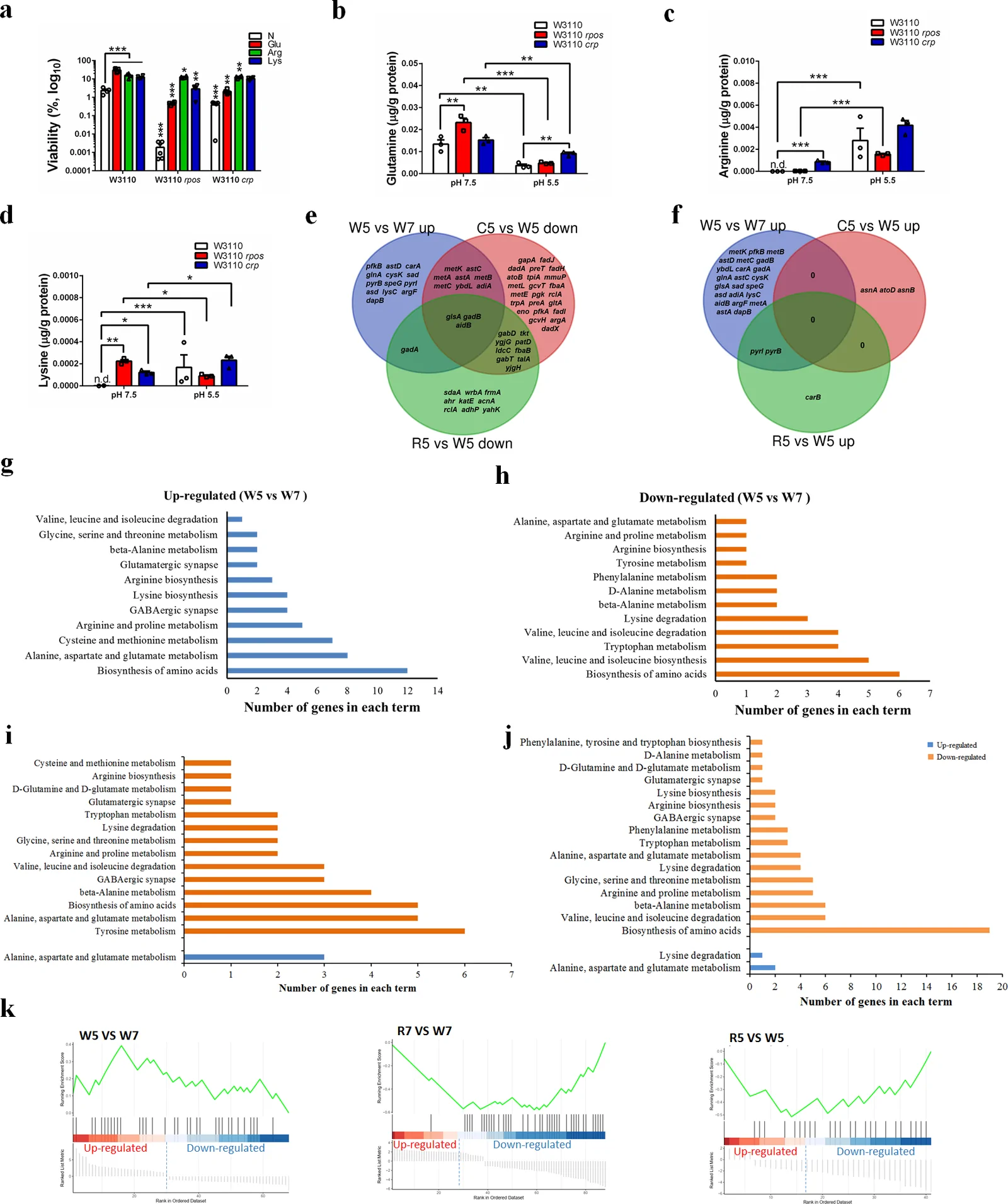
Roles of RpoS and Crp in regulating amino acid metabolism in AR under acidic conditions. (**a**) Exogenous amino acids increase the ARs of the W3110, W3110Δ*crp*, and W3110Δ*rpoS* strains. (**b**) Intracellular glutamate in the W3110, W3110Δ*crp*, and W3110Δ*rpoS* strains at pH 7.5 or 5.5. (**c**) Intracellular arginine in the W3110, W3110Δ*crp*, and W3110Δ*rpoS* strains at pH 7.5 or 5.5. (**d**) Intracellular lysine in the W3110, W3110Δ*crp*, and W3110Δ*rpoS* strains at pH 7.5 or 5.5. (**e**) Deletion of *crp* and *rpoS* leads to the downregulation of amino acid metabolism genes in acidic environments. (**f**) Upregulation of amino acid metabolism genes in acidic environments by deletion of *crp* and *rpoS*. (**g and h**) Enriched functions of upregulated genes (**g**) and downregulated genes (**h**) in wild-type W3110 at pH 5.5 compared with those at pH 7.5. (**i and j**) Upregulated (blue) and downregulated genes (orange) in (**i**) *rpoS-* and (**j**) *crp-*deficient strains after pH 5.5 adaptation compared to WT strains at pH 5.5. (**k**) Enrichment plots for the metabolic pathway gene sets that were strongly related to different pH in W3110, *rpoS-* and *crp-*deficient strains. Bars represent mean ± standard deviation (*n*  =  3–5). Bars with “*” are significantly different from controls as measured by one-way analysis of variance (*, *P* ≤ 0.05; **, *P* < 0.01; ***, *P* < 0.001).

As shown in Fig. 2a, Crp and RpoS modulate amino acid-dependent AR systems in *E. coli*. While exogenous glutamate enhanced survival of wild-type (WT) cells under pH 2.5 more effectively than arginine or lysine, this pattern reversed in *crp* and *rpoS* deletion strains, suggesting Crp and RpoS are critical for glutamate-mediated AR (Fig. 2a). Exogenous arginine and glutamate raised intracellular pH from 3.19 ± 0.18 to ~4.15–4.20 during pH 2.5 stress (Table 1). However, glutamate’s survival benefit in mutants lagged behind arginine (Fig. 2a), implicating Crp/RpoS-dependent mechanisms beyond pH homeostasis.

**TABLE 1:** Internal pH in acidic medium^*a*^

Strains		pHo = 5.5	pHo = 2.5		
			15 min	30 min	60 min
W3110	No addition	7.17 ± 0.09	4.13 ± 0.07	3.93 ± 0.05	3.19 ± 0.17
	+Glutamate	7.23 ± 0.05	4.58 ± 0.07	4.27 ± 0.02	4.2 ± 0.02
	+Arginine	7.19 ± 0.03	4.37 ± 0.01	4.24 ± 0.02	4.15 ± 0.02

^*a*^ pHo, medium pH.

Intracellular glutamate levels, although higher than other amino acids, declined significantly at pH 5.5 across all strains, consistent with glutamate decarboxylation activity buffering cytosolic pH (Fig. 2b, Table 1; Fig. S2a). Conversely, intracellular arginine increased in WT and mutants at pH 5.5 (Fig. 2c), while lysine accumulation diverged: elevated in WT and *crp* mutants but reduced in *rpoS* mutants (Fig. 2d).

RNA sequencing results further confirmed that pH 5.5 reshaped amino acid metabolism, with 25 genes upregulated and 17 downregulated (Fig. 2e and f; Table S4). Glutamate decarboxylase genes (*gadA*, *gadB*, *glsA*) were pH-inducible in WT but suppressed in mutants (Fig. 2e). *gadA* specifically required RpoS, while *glsA* required both regulators (23, 24). *gdhA* (glutamate dehydrogenase) expression dropped in mutants at pH 7.5, whereas *gabT* (GABA catabolism) (25) and *astC* (26) (glutamate synthesis) were Crp-dependent at pH 5.5.

Transcriptomic profiling revealed that condition-specific regulation of arginine metabolism components (Fig. 2e and f; Table S4). The decarboxylase gene *adiA* exhibited Crp-dependent repression, with Δ*crp* triggering ~3 fold (*P* = 1.01e−6) upregulation at pH 5.5. In contrast, *aidB* (isovaleryl-CoA dehydrogenase) was acid-inducible in WT but suppressed in Δ*crp* (pH 5.5) and Δ*rpoS* (pH 5.5/7.5), indicating dual regulatory control. The arginine synthesis operon *pyrI-pyrB* displayed RpoS-mediated repression, showing pH 5.5 induction in WT and further activation in Δ*rpoS* (Fig. 2f).

Metabolic perturbations extended beyond arginine pathways. While *ldcC* (lysine decarboxylase) expression increased in Δ*rpoS* strains across pH conditions (Table S4), its deletion did not alter AR phenotypes (Fig. S2b), suggesting functional redundancy. Conversely, Crp-dependent regulation of *metK* (methionine adenosyltransferase) proved critical (27), with Δ*metK* severely impairing pH 5.5 survival (Fig. S2c).

KEGG analysis revealed broad metabolic remodeling under acid stress. Butanoate metabolism, TCA cycle, and oxidative phosphorylation were suppressed in WT at pH 5.5, while GABAergic synapse and amino acid metabolism pathways were enriched (Fig. 2g; Data S1). Strikingly, Δ*crp* and Δ*rpoS* strains exhibited disproportionate downregulation of amino acid metabolic genes compared with WT (Fig. 2i and j). GSEA further delineated RpoS’s dominant role in metabolic adaptation, with 13 pH-inducible pathways in WT reduced to six in Δ*rpoS* (Fig. 2k).

Crp and RpoS coordinate AR through a tiered regulatory strategy: they hierarchically prioritize glutamate decarboxylation (*gad/gls* systems) over lysine/arginine-dependent pH buffering, reallocate metabolic resources by suppressing ATP-intensive processes (e.g., TCA cycle and oxidative phosphorylation), and deploy compensatory pathways (Δ*crp*-induced *adiA* or Δ*rpoS*-activated *pyrI-pyrB*) to maintain arginine homeostasis, revealing both division of labor and conditional redundancy in acid stress adaptation.

### RpoS and *crp* regulate nucleotide and energy metabolism under acidic conditions

**Fig 3:**
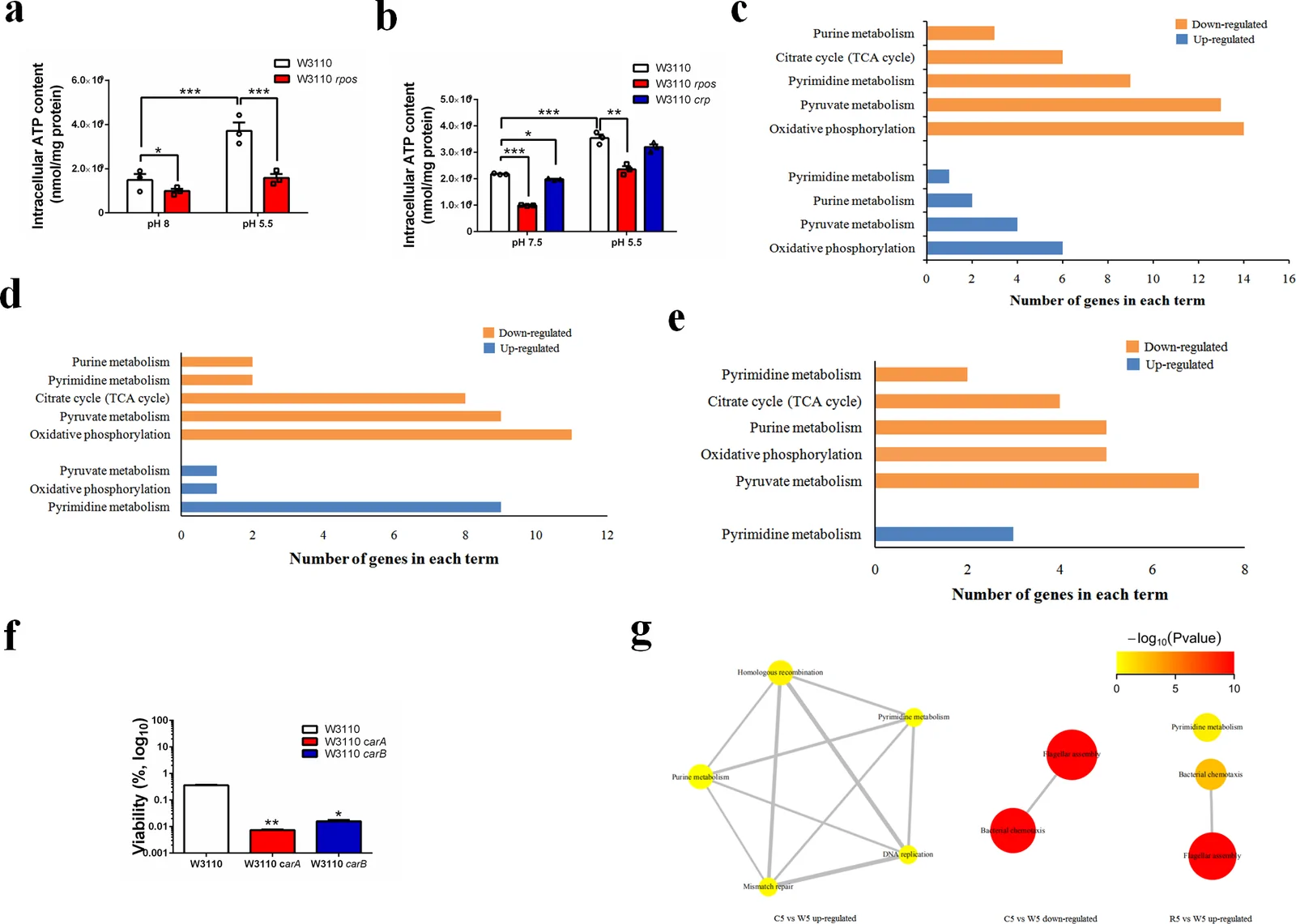
Intracellular ATP content in W3110Δ*crp* and W3110Δ*rpoS* mutants at different pH values. (**a**) Intracellular ATP content of the W3110Δ*rpoS* mutant at the stationary phase. (**b**) Intracellular ATP content of W3110Δ*crp* and W3110ΔrpoS mutants at the log phase. (**c**) Transcripts involved in nucleotide metabolism and the TCA cycle in W3110Δ*crp* compared with W3110 at pH 5.5. (**d**) Gene transcripts related to nucleotide metabolism and the TCA cycle in W3110 at different pH values. (**e**) Transcripts involved in nucleotide metabolism and the TCA cycle in W3110Δ*rpoS* compared with W3110 at pH 5.5. (**f**) Pyrimidine nucleotide synthesis pathway genes W3110Δ*carA* and W3110Δ*carB* are important for AR. (**g**) KEGG enrichment map of W3110, W3110Δ*crp,* and W3110Δ*rpoS* at acidic pH. Bars represent mean ± standard deviation (*n*  =  3). “*” are significantly different from controls as measured by one-way analysis of variance (*, *P* ≤ 0.05; **, *P* < 0.01; ***, *P* < 0.001).

Previous studies demonstrated that adenosine deamination enhances AR and that the purine biosynthesis pathway is critical for AR (18, 28). Here, we observed that *rpoS* deletion significantly reduced cellular ATP levels across different pH conditions (Fig. 3a and b). In contrast, the *crp* mutant exhibited decreased ATP levels at pH 7.5 but not at pH 5.5 (Fig. 3b). KEGG pathway analysis revealed that most oxidative phosphorylation genes were downregulated in the *crp* mutant (Fig. 3c and d; Table S5), while GSEA indicated upregulation of 11 glycolysis-related genes in *crp* deletions (Fig. S2d). These findings suggest that *crp* deletion sustains ATP levels under acidic conditions by enhancing glycolysis, the primary ATP synthesis pathway at low pH (29).

Additionally, *rpoS* deletion downregulated purine metabolism genes, including those involved in nucleoside degradation (Fig. 3e). Pyrimidine synthesis genes *carA* and *carB* were essential for acid survival (Fig. 3f), although their upregulation at low pH was independent of Crp or RpoS regulation.

Flagellar gene expression was modulated by *crp* and *rpoS* mutations as well as acidic pH. While flagellar assembly genes were generally downregulated at low pH (Fig. 3g), they remained highly expressed in the *rpoS* mutant at both pH 7.5 and 5.5 (Fig. S3). This suggests that RpoS suppresses motility genes under acidic conditions, possibly to conserve ATP. Conversely, Crp exhibited opposing regulatory effects on flagellar genes at low pH (Fig. 3g; Fig. S3).

### RpoS and *crp* modulate fatty acid metabolism to promote AR

**Fig 4:**
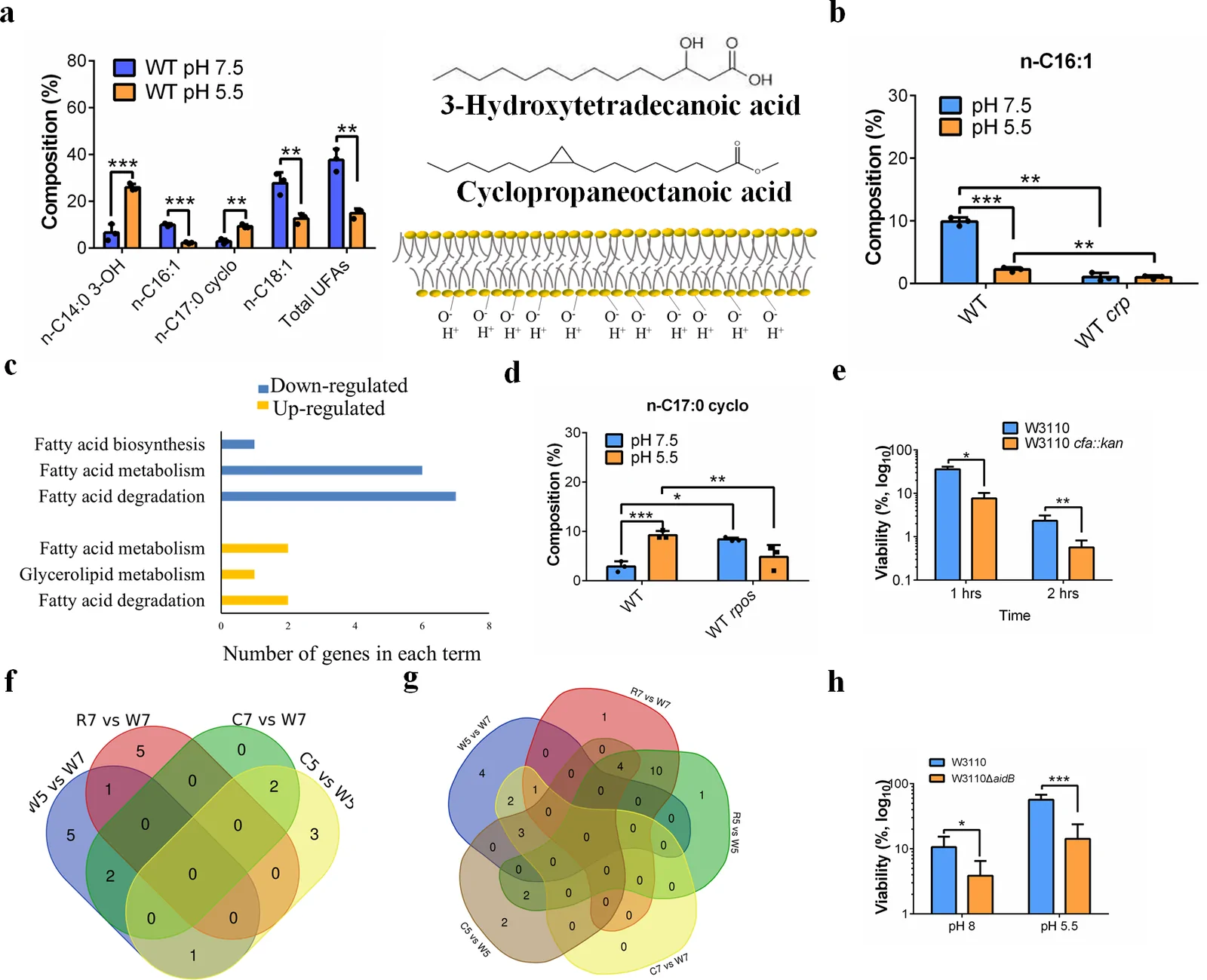
Fatty acid metabolism in W3110Δ*crp* and W3110Δ*rpoS* mutants at different pH values. (**a**) Fatty acid composition of W3110Δ*crp* and W3110Δ*rpoS* mutants at different pH values. (**b**) 9-Hexadecenoic acid regulated by Crp at both pH 7.5 and 5.5. (**c**) Transcripts of up- and downregulated fatty acid metabolism in W3110 at different pH values. (**d**) Cyclopropaneoctanoic acid regulated by RpoS at pH 7.5. (**e**) W3110Δ*cfa* mutation decreases the AR of *E. coli*. The AR was tested at pH 2.5. (**f**) Upregulated genes involved in fatty acid metabolism in W3110 and W3110Δ*rpoS* and W3110Δ*crp* deletions. (**g**) Downregulated genes involved in fatty acid metabolism in W3110 and W3110Δ*rpoS* and W3110Δ*crp* deletions. (**h**) The W3110Δ*aidB* mutation decreased the AR of *E. coli*. Bars represent mean ± standard deviation (*n*  =  3). “*” are significantly different as indicated, measured by one-way analysis of variance (*, *P* ≤ 0.05; **, *P* < 0.01; ***, *P* < 0.001).

Weak acidic pH altered fatty acid composition, enhancing bacterial survival. Levels of 3-hydroxytetradecanoic acid (n-C14:0 3-OH) and cyclopropaneoctanoic acid (n-C17:0 cyclo) increased at low pH in strain W3110 (Fig. 4a and Table 2), whereas 9-hexadecenoic acid (n-C16:1), oleic acid (n-C18:1), and total unsaturated fatty acids (UFAs) decreased. 3-Hydroxytetradecanoic acid, a lipopolysaccharide component (endotoxin) (30), may bolster membrane proton retention, reducing H^+^ influx (Fig. 4a).

**TABLE 2:** Fatty acid composition at different pHs in WT and mutants^*a*^

Fatty acids	Composition (%)					
	W3110		W3110 Δ*crp*		W3110 Δ*rpos*	
	pH 7.5	pH 5.5	pH 7.5	pH 5.5	pH 7.5	pH 5.5
n-C_14:0_	4.61 ± 0.15	3.20 ± 0.63	3.79 ± 1.37	4.33 ± 0.65	4.18 ± 1.80	2.51 ± 0.64
n-C_14:0_ 3-OH	6.60 ± 3.55	25.89 ± 1.52	21.14 ± 10.27	31.11 ± 8.64	42.73 ± 2.92	31.71 ± 6.29
n-C_16:1_	9.91 ± 0.65	2.24 ± 0.36	1.03 ± 0.66	1.01 ± 0.28	6.66 ± 2.65	3.05 ± 0.69
n-C_16:0_	41.55 ± 1.11	37.96 ± 2.32	40.20 ± 0.80	37.75 ± 5.35	35.07 ± 7.03	36.37 ± 7.46
n-C_17:0_ cyclo	3.38 ± 0.75	9.25 ± 0.83	6.94 ± 4.64	7.86 ± 3.24	8.45 ± 0.50	6.46 ± 1.39
n-C_18:1_	27.73 ± 4.60	12.62 ± 2.13	26.01 ± 8.95	16.39 ± 2.55	21.22 ± 8.16	11.72 ± 2.77
n-C_18:0_	7.12 ± 1.17	8.85 ± 2.54	8.17 ± 2.07	5.12 ± 2.49	3.76 ± 0.88	8.17 ± 4.08
Total UFAs	37.63 ± 4.76	14.86 ± 2.03	26.68 ± 8.71	17.29 ± 2.25	27.88 ± 10.67	14.78 ± 2.37

^*a*^ n-C14:0, Myristic acid; n-C14:0 3-OH; 3-Hydroxytetradecanoic acid; n-C16:1, 9-Hexadecenoic.

Crp mutation reduced 9-hexadecenoic acid levels at both pH 7.5 and 5.5 (Fig. 4b), likely due to downregulation of fatty acid degradation genes (*fadJ*, *fadH*, *fadI*), which are linked to β-oxidation and biofilm formation (Fig. 4c; Table S6). *rpoS* deletion decreased cyclopropaneoctanoic acid production at pH 5.5 (Fig. 4d) by suppressing *cfa*, a key cyclopropane synthase gene (Table S6). *cfa* deletion severely impaired bacterial survival at pH 2.5 (Fig. 4e), consistent with its role in extreme acid tolerance (31).

KEGG analysis showed broad downregulation of fatty acid biosynthesis genes at low pH (Fig. 4c). *aidB*, regulated by RpoS (32), mitigates DNA damage and generates N-acylhomoserine lactone (AHL), a quorum-sensing signal (33, 34). *aidB* was upregulated at acidic pH and *rpoS*-dependent (Data S1), with Crp further influencing its expression. *aidB* deletion markedly impaired AR even after pH 5.5 adaptation (Fig. 4h).

Collectively, RpoS and Crp coordinate fatty acid metabolism and membrane remodeling to optimize AR.‌

### ‌Chaperones, membrane proteins, and transporters regulated by RpoS and crp contribute to AR

Chaperone proteins are crucial for maintaining cellular homeostasis under both normal and stress conditions (35). Under acidic pH (5.5), we observed differential regulation of 13 chaperones, with all except *fliJ* being upregulated (Table S7). Notably, *hdeA* and *hdeB*, previously implicated in AR (36, 37), were among the upregulated chaperones, reinforcing their importance in AR.

**Fig 5:**
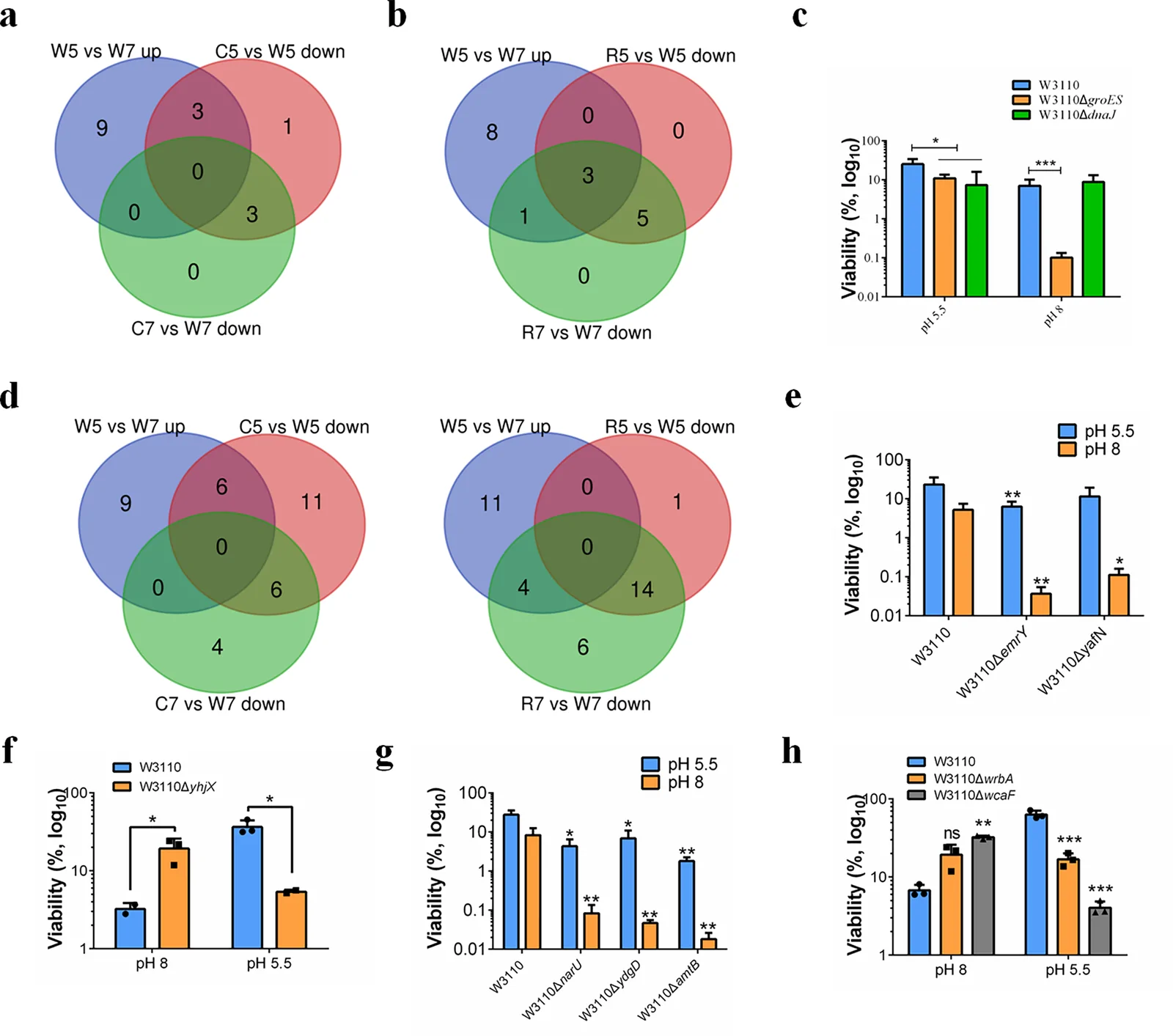
Chaperones, membrane proteins, and transporters regulated by RpoS and Crp are important for AR. (**a**) Chaperon regulated by Crp at pH 7.5 and 5.5. (**b**) Chaperon regulated by RpoS at pH 7.5 and 5.5. (**c**) Chaperons W3110Δ*groES* and W3110Δ*dnaJ* deletions decrease the stationary-phase AR of *E. coli* under different pH conditions. (**d**) Venn diagram of membrane proteins regulated by Crp and RpoS at acidic pH. (**e**) W3110Δ*emrY* and W3110Δ*yafN* play a role in AR. (**f**) W3110Δ*yhjX* plays a role in AR. (**g**) Transporters and other genes are required for AR. (**h**) W3110Δ*wrbA* and W3110Δ*wcaF* play a role in AR. Bars represent mean ± standard deviation (*n*  =  3). “*” are significantly different from controls as measured by one-way analysis of variance (ns, not significant; *, *P* ≤ 0.05; **, *P* < 0.01; ***, *P* < 0.001).

Crp deletion led to the downregulation of *cusF*, *cbpM*, and *cbpA* at acidic pH (38) (Fig. 5a; Table S7). Conversely, *cbpM*, *cbpA*, *hdeB*, and *hdeA* were upregulated under acidic conditions but suppressed upon *rpoS* deletion (Fig. 5B; Table S7). While *fliJ* expression was reduced by both weak acid stress and Crp loss, *rpoS* mutation had an opposing effect, increasing *fliJ* levels compared with wild-type and *crp*-mutant strains (Fig. S1j). Seven of the 13 pH-responsive chaperones, including *clpB*—a known acid survival factor (39)—remained unaffected by *crp* or *rpoS* deletion. Functional analyses revealed that *groES* mutation impaired stationary-phase extremely acidic survival at both pH 8 and 5.5 growth conditions (Fig. 5c), whereas *dnaJ* deletion specifically compromised AR at pH 5.5 (Fig. 5c). Loss of *ibpA* had a minor but detectable effect on viability at pH 8 (Fig. S4a). However, all mutations *groES*, *dnaJ,* and *ibpA* reduce growth at acidic pH (Fig. S4b).

Among 22 membrane-associated genes differentially expressed at pH 5.5 (15 upregulated, 7 downregulated; Table S8), six Crp-dependent upregulated genes—including *cusB* and *cusC*, components of the Cu (+)/Ag (+) efflux RND transporter—were suppressed in *crp* mutants (Fig. 5d; Table S8).

Transporters also played a critical role in AR. The multidrug efflux MFS transporter EmrY was significantly induced at pH 5.5 (40), and its deletion markedly reduced stationary-phase survival (Fig. 5e). Another MFS transporter, YhjX, was downregulated by Crp at pH 5.5 (Table S8). Intriguingly, *yhjX* mutation enhanced viability at pH 8 but impaired AR at pH 5.5 (41) (Fig. 5f). The type I toxin-antitoxin system component YafN was essential for AR only at pH 5.5 (Fig. 5e). The acid shock protein Asr, upregulated independently of Crp or RpoS, was required for survival at pH 5.5 (Fig. S4c), consistent with prior findings (42). Additionally, nitrate/nitrite transporter NarU, serine protease YdgD, and ammonium transporter AmtB contributed to AR, with their deletion exerting a stronger inhibitory effect at pH 8 than at pH 5.5 (Fig. 5g).

Notably, *wrbA* (quinone oxidoreductase) and *wcaF* (acetyltransferase), both upregulated by Crp and/or RpoS at acidic pH (Data S1), exhibited pH-dependent survival effects: their loss reduced viability at pH 2.5 (following pH 5.5 adaptation) but enhanced survival at pH 8 (Fig. 5h). Furthermore, Crp-mediated upregulation of *argT* and *rutB* contributed to AR at pH 5.5 (Fig. S4d).

These findings underscore the complex interplay between chaperones, membrane proteins, and transporters in bacterial AR, with Crp and RpoS serving as key regulatory nodes in pH-dependent stress adaptation.

### Weak acid adaptation reveals pH-dependent antibiotic potentiation through RpoS/Crp regulatory networks

**Fig 6:**
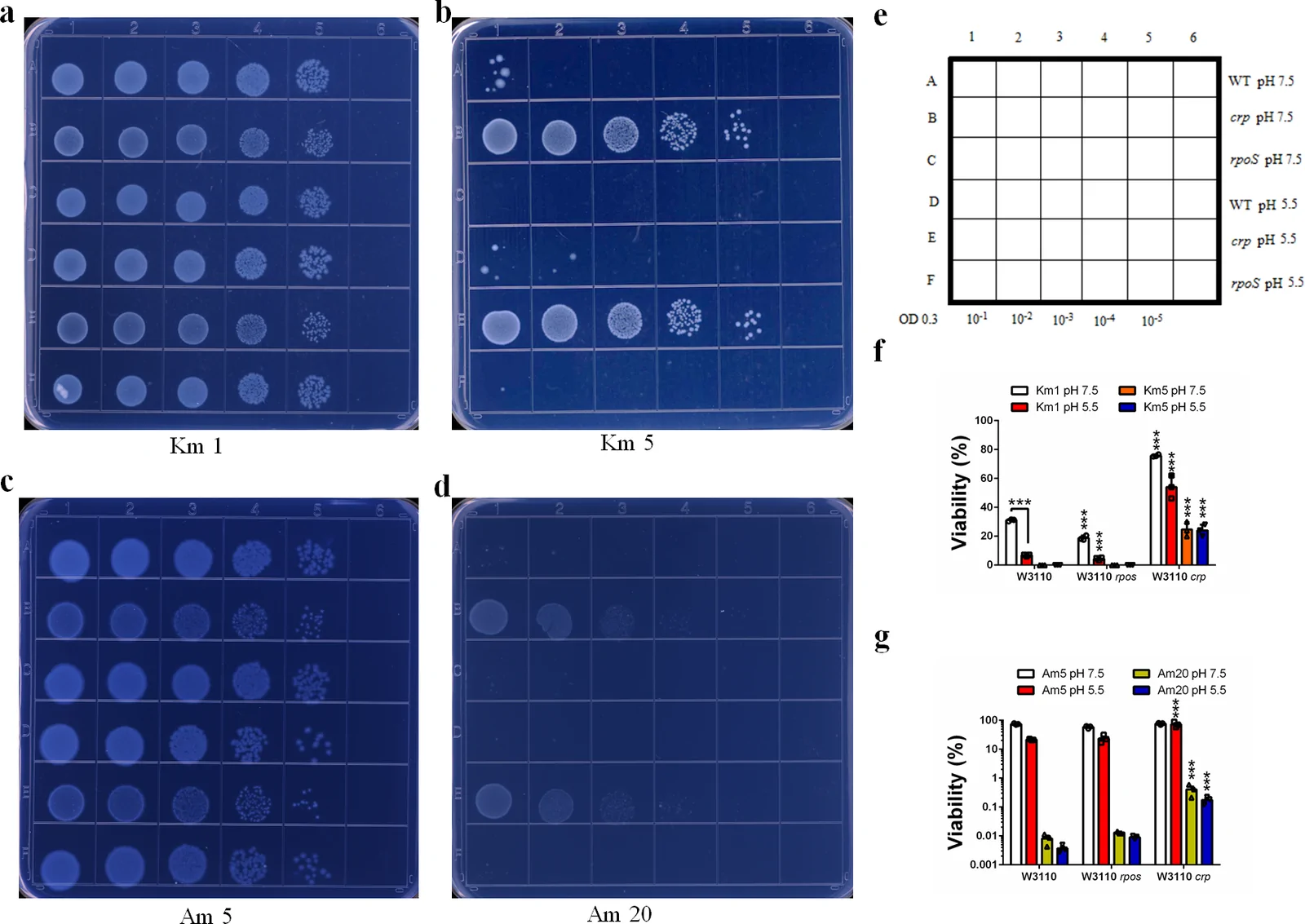
Effects of antibiotics on the survival of bacteria at different pH values. (**a and b**) Kanamycin resistance of W3110 and its mutants after different pH treatments. Km 1, concentration of kanamycin is 1 μg/mL; Km 5, concentration of kanamycin is 5 μg/mL. (**c and d**) Ampicillin resistance of W3110 and its mutants after different pH treatments. Am 5, concentration of ampicillin is 5 μg/mL; Km 5, concentration of ampicillin is 20 μg/mL. (**e**) Schematic diagram of the experimental process of panels **a–d**. After the wild-type and mutant strains were cultured to OD6_00_ ~ 0.3 at different pH values, the same amount of bacteria was inoculated onto plates with different concentrations of antibiotics, and growth was checked after overnight incubation. (**f**) Survival of W3110 and its mutants after incubation at different pH values following Km treatment. (**g**) Survival of W3110 and mutant plants after Amp treatment at different pH values. Bars represent mean ± standard deviation (*n*  =  3). “*” are significantly different from controls as measured by one-way analysis of variance (***, *P* < 0.001).

To investigate how acid adaptation influences antibiotic resistance, we examined Δ*rpoS* and Δ*crp* mutants under varying pH conditions with antibiotic treatment. Deletion of *rpoS* significantly reduced kanamycin resistance at both pH 7.5 and 5.5 (Fig. 6a, b, e and f), while showing no effect on ampicillin susceptibility (Fig. 6c through e and g). In contrast, *crp* deletion significantly increased anti-kanamycin and anti-ampicillin activity at both pH 7.5 and 5.5, especially at pH 5.5 (Fig. 6).

These findings suggest that the combination of antibiotics and AR genes as targets may be a potential method for treating pathogenic bacterial infections.

## DISCUSSION

**Fig 7:**
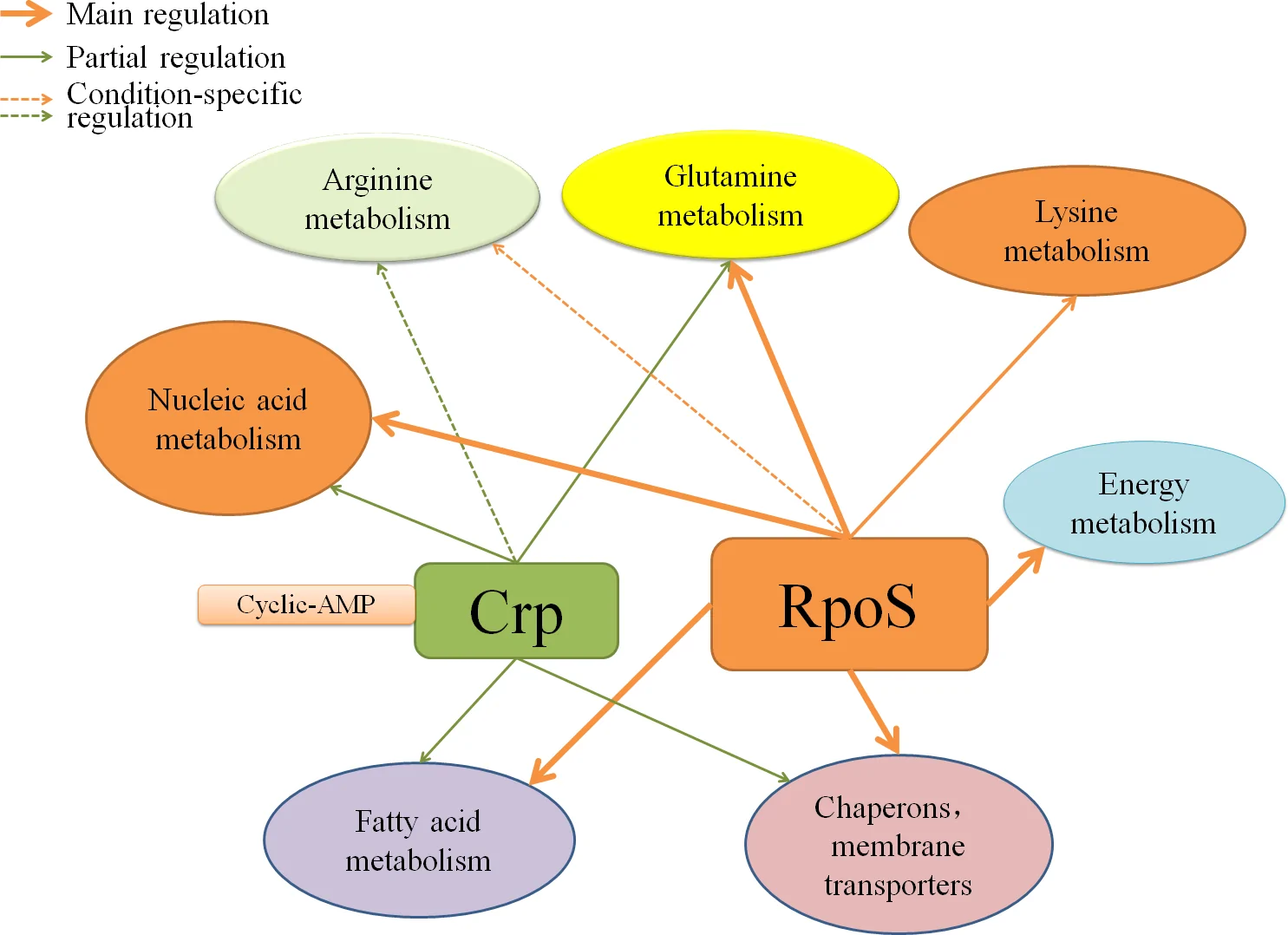
Profiles of metabolism regulated by Crp and RpoS at different pH values. The thickness of the lines represents the degree of regulation.

Our systematic analysis reveals that the AR response in *E. coli* is coordinately regulated by the global regulators Crp and RpoS through distinct yet overlapping metabolic pathways (Fig. 7). RpoS-mediated AR involves a multiplex system encompassing amino acid metabolism (glutamine, lysine, and arginine), energy homeostasis, fatty acid metabolism, and functional modulation of chaperones, membrane proteins, and transporters. Crp similarly contributes to AR by regulating overlapping pathways, with differential pH-dependent effects. Notably, we identified additional RpoS- and Crp-independent regulatory systems that further facilitate AR adaptation.

The rumen’s weakly acidic environment (pH ~5.5–6.5) in cattle (11, 13) may precondition *E. coli* O157 for gastric survival, elevating the risk of foodborne transmission (11). Our findings demonstrate that Crp and RpoS govern pH-dependent amino acid metabolism: (i) glutamine decarboxylation is dually regulated by both regulators at neutral and acidic pH; (ii) arginine synthesis requires Crp/RpoS exclusively at pH 7.5; whereas (iii) lysine metabolism is RpoS-dependent only under acidic conditions. These stratified regulatory mechanisms suggest niche-specific optimization of amino acid utilization for AR.

ATP pools emerged as critical determinants of AR (18). *rpoS* deletion downregulated TCA cycle and oxidative phosphorylation genes under acidic stress, reducing ATP levels and potentiating kanamycin susceptibility. In contrast, *crp* deletion maintained ATP homeostasis at low pH, correlating with sustained antibiotic tolerance. This dichotomy implies that ATP availability modulates AR efficacy in a regulator-specific manner.

Fatty acid metabolism contributes to AR through dual mechanisms: (i) acidic pH downregulated β-oxidation genes, limiting ATP synthesis via fatty acid catabolism (43) while (ii) inducing production of 3-hydroxytetradecanoic and cyclopropaneoctanoic acids—membrane modifiers that may restrict H^+^ influx. Biofilm-related and quorum-sensing genes were similarly pH-sensitive, suggesting extracellular matrix remodeling as an auxiliary AR strategy (44, 45).

Chaperones, membrane proteins, and transporters proved essential for AR (46, 47), with Crp/RpoS regulating their activity to maintain intracellular homeostasis. Intriguingly, acidic pH upregulated two-component systems that were suppressed in *crp* mutants (Fig. S4e through g), and their functional relevance to AR warrants further investigation.

This work delineates the AR regulatory network in *E. coli*, highlighting potential targets for the development of novel antimicrobial agents. Conversely, engineered strains harnessing these AR mechanisms could potentially enhance bioremediation efficiency in acidic industrial wastewater (48). Future studies should explore interspecies conservation of these pathways and their therapeutic applicability.

## MATERIALS AND METHODS

### Bacterial strains and culture media

The plasmids and bacterial strains used in this study are listed in Table S9. Minimal E medium (49) containing 0.4% glucose (EG) with a pH between 7.5 and 7.0 (adjusted by NaOH) or a pH between 5.5 and 2.5 (adjusted by HCl) was used for bacterial culture. Luria–Bertani broth (LB) with either 100 mM morpholinepropanesulfonic acid (MOPS) (pH = 8) or 100 mM morpholine- ethanesulfonic acid (MES) (pH = 5.5) was used for the AR test. Antibiotics were used at the following concentrations: ampicillin, 100 mg/mL; kanamycin, 25 mg/mL; and chloramphenicol, 30 mg/mL. The growth curves of *E. coli* strains were measured by Bioscreen C (Oy Growth Curves Ab Ltd).

### One-step inactivation of chromosomal genes in *E. coli* and P1 transduction

*E. coli* knockout mutants were a gift from Prof. Hiroshi Kobayashi (Chiba University, Japan) and constructed via conventional Red-mediated recombination (50). Linear fragments containing resistance genes and 60 homologous sequence pairs on both sides of the target gene were introduced into *E. coli* BW25113 carrying the helper plasmid pKD46 carrying Red recombinase through electroporation to replace the target gene (Table S9). The primers used to amplify these genes are shown in Table S10. L-arabinose (1 mM), kanamycin, and chloramphenicol were added to the medium to select the positive mutants after electroporation. The DNA from each individual transformant was isolated, and the integration of the resistance gene was confirmed via PCR amplification. The Red helper plasmid pKD46 was cured by growth overnight at 42°C (51).

The target knockout genes were transferred from BW25113 to W3110 via P1 phage, as previously described (52). Culture recipient bacteria to logarithmic phase at 37°C, treat with 5 mM CaCl₂ to enhance P1 phage adsorption, then incubate with serially diluted P1 lysate (10⁻¹–10⁻³) at 37°C for 20 min; after centrifugation (3,500 rpm, 15 min), resuspend pellet in sterile saline, plate 0.1 mL on selective media, and incubate at 37°C for 48 h to count transductants, alongside an uninfected control to assess background. Transformation was performed as described previously (53, 54). Plasmid DNA is adsorbed onto the surface of competent cells through an ice bath, and DNA uptake is promoted after a brief heat shock at 42°C. The transductants of W3110 were selected by kanamycin or chloramphenicol.

### AR test

AR was measured in cells grown in the logarithmic growth phase, as described previously (29, 55), with the following modifications. The wild-type cells and mutants were cultured overnight in LB with or without antibiotics. The cells were subsequently diluted 1,000-fold with EG medium at pH 7.5 and cultured at 37°C until the optical density at 600 nm (OD_600_) reached 0.3. After harvested, cells were suspended in a twofold volume of EG medium at pH 5.5, and incubated without shaking for 3 h for acid adaptation. For the AR test, the adapted cells were challenged with EG medium with a pH of 2.5 at 37°C for 1 or 2 h. Then, they were diluted with phosphate-buffered saline (PBS) and plated on LB agar plates. After culture at 37°C overnight, the surviving colonies were counted.

The AR1 assay in the stationary phase was performed according to previously reported methods (16, 56). The cells were cultured overnight in LB with or without antibiotics. Overnight cultures were transferred (1:1,000 dilution) to 4 mL of fresh LB buffered with MOPS or MES at a pH of 8 or a pH of 5.5 in test tubes with shaking (220 rpm) at 37°C to the stationary phase (22 h), followed by a 1:1,000 dilution into prewarmed EG (37°C, pH = 2.5). Cell viability was determined after 2 h at pH 2.5 by counting the surviving colonies cultured on LB agar plates at 37°C overnight.

### RNA extraction and RNA-seq analysis

The cells were cultured at pH 7.5 in EG medium until the OD_600_ reached 0.3–0.4. The cell pellet was harvested and resuspended in an equal volume of fresh EG medium at either pH 7.5 or 5.5. After incubation for 1 h, total RNA was isolated via RNA-Solv reagent (Omega) according to the manufacturer’s protocol. The concentration and purity of the RNA were determined via the GeneQuant RNA/DNA Calculator (Pharmacia-Biotech, Cambridge, UK). The isolated RNA was sequenced on an Illumina HiSeq 2000. The cleaned reads were aligned to the reference sequences in the National Center for Biotechnology Information (NCBI) database. Gene expression levels are represented by the reads per kilobase per million reads (RPKM) method, as described by Kojima and colleagues (57). The RNA-seq analyses were repeated in three independent experiments with three biological replicates. Differentially expressed genes (DEGs) were identified via the DEGseq package, with a cutoff of a fold change >2 and a *P*-value < 0.01.

### Quantitative polymerase chain reaction (qPCR)

qPCR was conducted with gene-specific primers on a CFX96 Real-Time System (Bio-Rad, USA). The primer sequences, sourced from Sangon (Guangzhou, China), are provided in Table 3. Analyses were performed in duplicate across three biological replicates per experiment, following established protocols (58). Differential expression was determined using thresholds of a fold change >2 and a *P*-value < 0.01.

**TABLE 3:** Primers used in qPCR

Primer name	Sequence (5′→3′)
aldB qPCR F	CTGTACTGCTGCTAATGGAA
aldB qPCR R	CCGGAATAATGTTTTGCGTT
aroM qPCR F	CTGACGGAATACATTGACGA
aroM qPCR R	TTATCGAGCACTTCAACCAC
bsmA qPCR F	TGTTGATGTTAAGTGCCTGT
bsmA qPCR R	ACAGCTTTCGCTTTGATTTC
fliJ qPCR F	ATCAGCAGTTTATCCAGACG
fliJ qPCR R	TCTTTTTCTGATCGAGGCG
gspF qPCR F	CAACACTCCACACTAACAGT
gspF qPCR R	TTAAACATCTGCGCTTGTTG
ydcS qPCR F	GAACCGCCTACCAATTTAGA
ydcS qPCR R	CTGACCATTTCATCGGAAGT
ydcT qPCR F	ATCAACGTAAACCGTCACAA
ydcT qPCR R	GTGATACCGAGAGACTGTTG
yjcB qPCR F	GTGCCGTAATGATGTTTCTG
yjcB qPCR R	TCTCTTCTATCATCACCGACT
rrsB qPCR F	TTGCTCATTGACGTTACCC
rrsB qPCR R	CCAGTATCAGATGCAGTTCC

### Gene ontology annotation and KEGG pathway analysis

Gene Ontology (GO) comprises three aspects to describe gene functions: BP (biological process), MF (molecular function), and CC (cellular component). The Kyoto Encyclopedia of Genes and Genomes (KEGG) Pathway database was used as a reference for mapping the molecular interactions and reaction and relation networks for metabolism (59). The online web tool Database for Annotation, Visualization, and Integrated Discovery (DAVID) (29, 60) was used for functional enrichment analysis of GO terms via the KEGG pathways. The EASE score was applied to evaluate whether the DEGs were significantly enriched for a specific gene function. The Benjamini‒Hochberg (BH) method was used to adjust *P* values for multiple comparisons, and *P* < 0.01 was considered significant.

Gene Set Enrichment Analysis (GSEA) was used to identify pathways enriched in ranked gene lists (61, 62); software and packages were from the MSigDB database (http://software.broadinstitute.org/gsea/downloads.jsp). The pH-dependent gene expression profiles and key pathways were analyzed, and the enriched pathways with *P*  <  0.01 were chosen for further analysis. The KEGG Pathway database was chosen as the back-end database of GSEA. The results of the GSEA of different expression profile data sets were intersected to obtain through the core enrichment (61) of KEGG pathways strongly related to metabolic pathways.

### Measurement of ATP content

The intracellular ATP content was measured, as described previously (18). After being cultured as described in “AR Test,” the cells were harvested by centrifugation at 10,000 × *g* at 4°C for 5 min. The cells were digested with lysis buffer containing 20 mM glycine, 50 mM MgSO_4_, 4 mM EDTA, and 50% methanol at 70°C for 30 min and then centrifuged at 10,000 × *g* for 5 min at 4°C. The ATP content in the supernatant was immediately measured via a luminometer (Turner Designs, CA, USA) with luciferase (Sigma‒Aldrich, USA). The ATP concentrations were calculated via the ATP standards included in the experiment.

### Analysis of amino acid composition

Different strains were cultivated to OD_600_ = 0.3 in 20 mL of LB medium, and then, the strains were harvested and incubated in fresh EG media at pH 7.5 and 5.5 for 3 h. After separation and purification, the concentration of proteins was measured by absorption at 595 nm using the Bradford Protein Assay (BioRad, Bradford), with bovine serum albumin as the standard. The concentration of free amino acids was determined via high-performance liquid chromatography (HPLC). Single amino acid solutions (Sigma‒Aldrich, USA) were prepared separately as standards. The proteins of the strains were precipitated from each sample by adding 200 μL of trifluoroacetic acid (TFA) or methanol solution (volume ratio  =  1:10) and centrifuged at 20,000 × *g* for 10 min. The free amino acids were then eluted from the sample supernatants by a mixture of sodium acetate, methanol, or triethanolamine (TEA) solution (volume ratio  =  2:2:1) or a mixture of methanol, water, TEA, or phenylisothiocyanate (PITC) solution (volume ratio  =  7:1:1:1). The samples were separated via an HPLC system (Thermo Fisher Scientific, Minneapolis, Minnesota, USA). The data were analyzed via Clarity Lite software (63), and all samples and standards were analyzed at least in triplicate.

### Analysis of fatty acid composition

Cellular lipids were measured via gas chromatography–mass spectrometry (GC‒MS), as previously reported (64). The cells were cultured as described in “Analysis of amino acid composition.” Cellular lipids were saponified at 90°C by adding 1 mL of 1 M NaOH in methanol:H_2_O (1:1 by volume). The samples were incubated in an incubator with shaking for half an hour. Fatty acids were methylated by the addition of 2 mL of 6 M HCl in methanol. The samples were heated at 80°C for 10 min and cooled to −20°C immediately. The fatty acid methyl esters were extracted with 1.25 mL of petroleum ether, and this process was repeated three times. The extracted samples were dried under a stream of nitrogen in a fume hood. The extracted esters were analyzed via GC‒MS (30). The results were interpreted as percentages of the total fatty acids and represented as the mean ± standard deviation from three independent determinations.

### Analysis of nucleotide composition

The nucleotide composition was analyzed by HPLC using 25 mM KH_2_PO_4_ in methanol as the mobile phase. UV absorption was detected at a wavelength of 254 nm (65). The standard curve for HPLC analysis was generated from standard CTP, UTP, GTP, and IMP at concentrations ranging from 10 ng to 100 μg.

### Measurement of the intracellular pH

The intracellular pH (pHi) was determined by the distribution of salicylic acid between the outside and inside of the cells, as described previously (18). After being cultured in EG medium at pH 5.5 for 3 h, the cells were harvested and resuspended in EG medium at pH 5.5 or 2.5 at 1 × 10^9^ cells/mL, after which ^[14C]^salicylic acid (10 µM, 0.2 µCi/mL) was added. After the mixture was incubated at 37°C for 15 min, 1 mL of the medium was centrifuged at 10,000 × *g* for 5 min through a 0.2-mL oil mixture (laurylbromide and liquid paraffin). The radioactivity of the supernatant and the resuspended pellet was measured to obtain the indicator concentrations outside and inside the cells, respectively. The amounts of protein in the pellets were measured, and the radioactivity of the pellet was divided by the internal water content calculated from the protein content of the pellet. The pHi was calculated via the following equation:

pHi = log((*A*_in_/*A*_out_)(10^pKa^ + 10^pHout^) − 10^pKa^), where *A*_in_ and *A*_out_ are the concentrations of salicylic acid inside and outside of cells, respectively, and pH_out_ is the medium pH. The pKa of salicylic acid is 2.89.

### Statistical analysis

All measurements were repeated three times from separate cultures. The data are presented as the means ± standard deviations of samples from two or three independent experiments. The statistical significance of differences between means was determined via unpaired or paired two-tailed Student’s *t*-tests. A *P-*value < 0.05 was considered statistically significant. One-way ANOVA followed by Bonferroni post-hoc correction or the F test was used to determine significant differences (*P* < 0.05) between groups (66).

## SUPPLEMENTAL MATERIAL

Supplemental figures spectrum.02063-25-s0001.docx Fig. S1 to S4.

Supplemental material spectrum.02063-25-s0002.xlsx Additional experimental details-genes expression profiles.

Supplemental tables spectrum.02063-25-s0003.xlsx Tables S1 to S3.

Table S4 spectrum.02063-25-s0004.xlsx KEGG amino acid.

Table S5 spectrum.02063-25-s0005.xlsx KEGG nuclear.

Table S6 spectrum.02063-25-s0006.xlsx Kegg analysis of fatty acid metabolism genes regulated by Crp and RpoS deletions compared with W3110 at pH 5.5 and 7.5.

Table S7 spectrum.02063-25-s0007.xls Chaperones regulated by different pH in W3110.

Table S8 spectrum.02063-25-s0008.xlsx Membrane proteins and transporters regulated by different pH in W3110.

Supplemental tables spectrum.02063-25-s0009.docx Tables S9 and S10.

ASM does not own the copyrights to Supplemental Material that may be linked to, or accessed through, an article. The authors have granted ASM a non-exclusive, world-wide license to publish the Supplemental Material files. Please contact the corresponding author directly for reuse.
