# Supplementary material for: Quantitative transcriptomic and metabolic analyses reveal the roles of RpoS and Crp in the acid resistance system 1 in Escherichia coli
Source: Microbiol Spectr. 2026 Jun 15;14(7):e02063-25. doi: 10.1128/spectrum.02063-25 (PMC13339887; doi:10.1128/spectrum.02063-25)
Supplement: Supplemental figures — Fig. S1 to S4. [file spectrum.02063-25-s0001.docx]

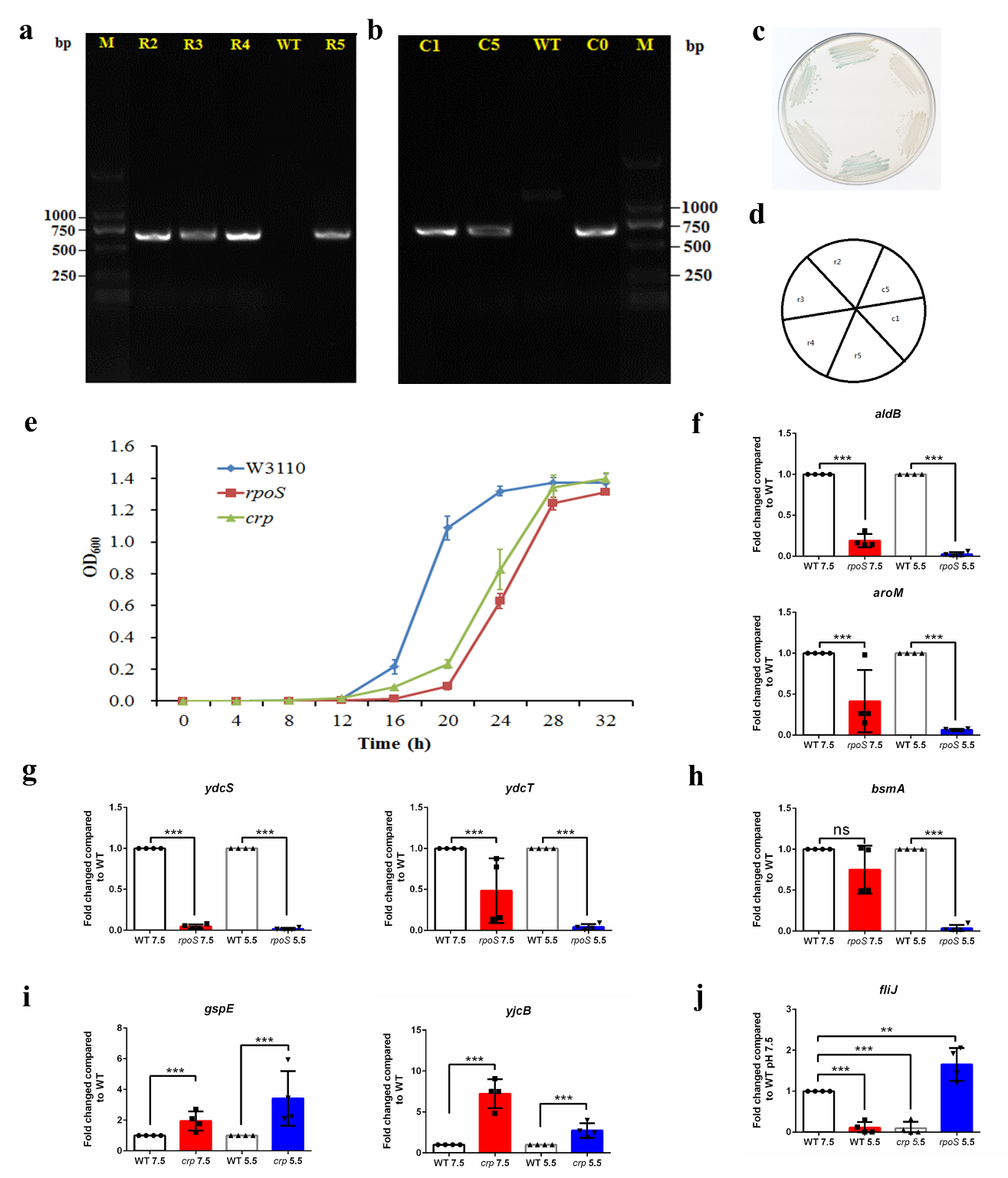


**Supplementary Fig. 1. Construction and test of *crp* and *rpoS* deletions and qPCR analysis. a-b** Detection of *crp* and *rpoS* gene knockout by polymerase chain reaction. M, DNA marker DL2000; WT, W3110; R2-R4, W3110Δ*rpoS*; R5, BW25113Δ*rpoS*; C1 and C5, W3110Δ*crp*, and C0, BW25113Δ*crp*. **c-d** Expression detection of lac operon induced by x-GAL. Mutants grow on LB plants with x-GAL. Symbols are as described above (r, W3110Δ*rpoS*, c, W3110Δ*crp*). **e** Growth curves of W3110Δ*crp* and W3110Δ*rpoS* at pH 7.0 in EG medium. **f** *aldB* and *aroM* expression in W3110 (WT) and W3110Δ*rpoS* (*rpoS*) at pH 7.5 (7.5) and pH 5.5 (5.5). **g** *ydcS* and *ydcT* expression in WT and *rpoS* at different pH. **h** *bsmA* expression in WT and *rpoS* at different pH. **i** Expression of *gspE* and *yjcB* in WT and *rpoS* at different pH. **j** Expression of *fliJ* at pH 5.5 in WT and mutants. Bars represent mean ± standard deviation (n = 3-4/group, One-way ANOVA, ns, not significant; **, p< 0.01; ***, p< 0.001).


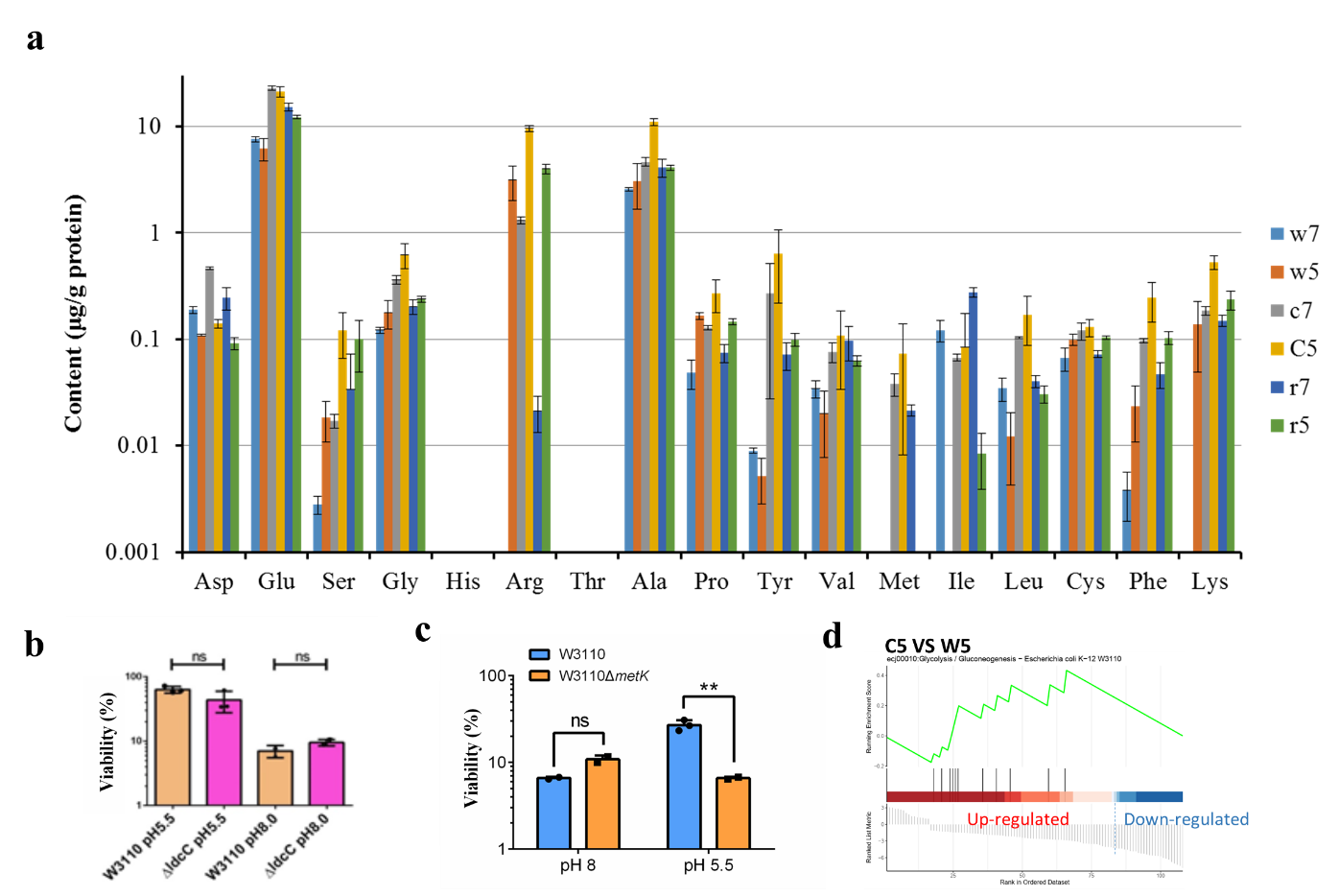


**Supplementary Fig. 2 a** Intracellular free amino acid content. W7, wild type W3110 treated with EG medium at pH 7.5; W5, wild type W3110 treated with EG medium at pH 5.5; C7, W3110Δ*crp* deletion treated with EG medium at pH 7.5; C5, W3110Δ*crp* deletion treated with EG medium at pH 5.5; R7, W3110Δ*rpoS* deletion treated with EG medium at pH 7.5; R5, W3110Δ*rpoS* deletion treated with EG medium at pH 5.5; N.D., not detected. **b** *ldcC* gene do not affect acid resistance. **c** *metK* play a role in acid resistance. **d** Enrichment plots for the glycolysis metabolic pathway gene sets related to acidic pH in W3110 and *crp*-deficient strains. Bars represent mean ± standard deviation (n = 3/group). ns, not significant; **, p< 0.01.


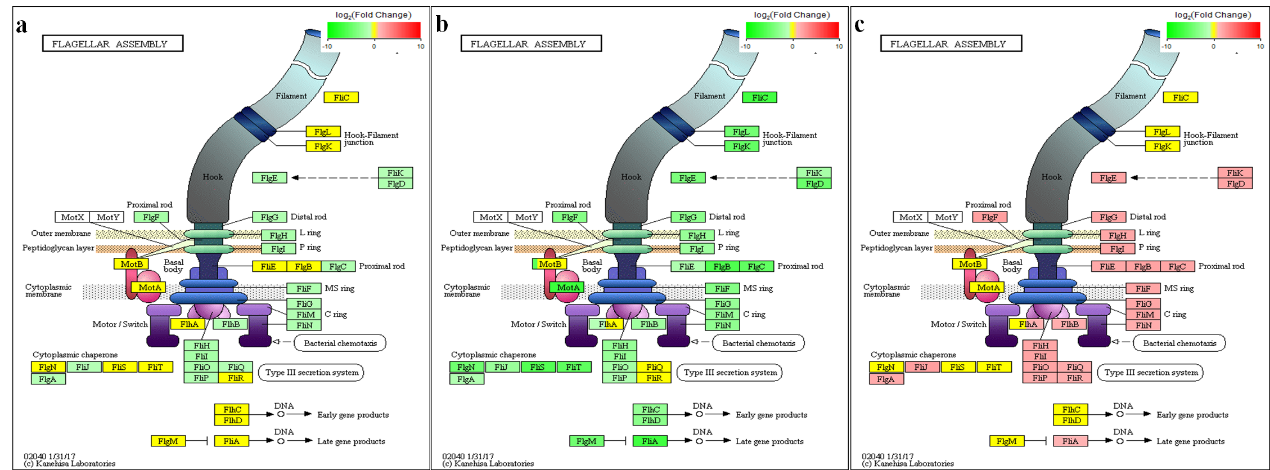


**Supplementary Fig. 3 Flagellar assembly in wt and mutants at different pH**. **A.** Genes expression of flagellar assembly in wild type at pH 5.5 compared to pH 7.5. **B.** Genes expression of flagellar assembly in *crp* mutant at pH 5.5 compared to wild type W3110. **C.** Genes expression of flagellar assembly in *rpoS* mutant at pH 5.5 compared to wild type W3110.


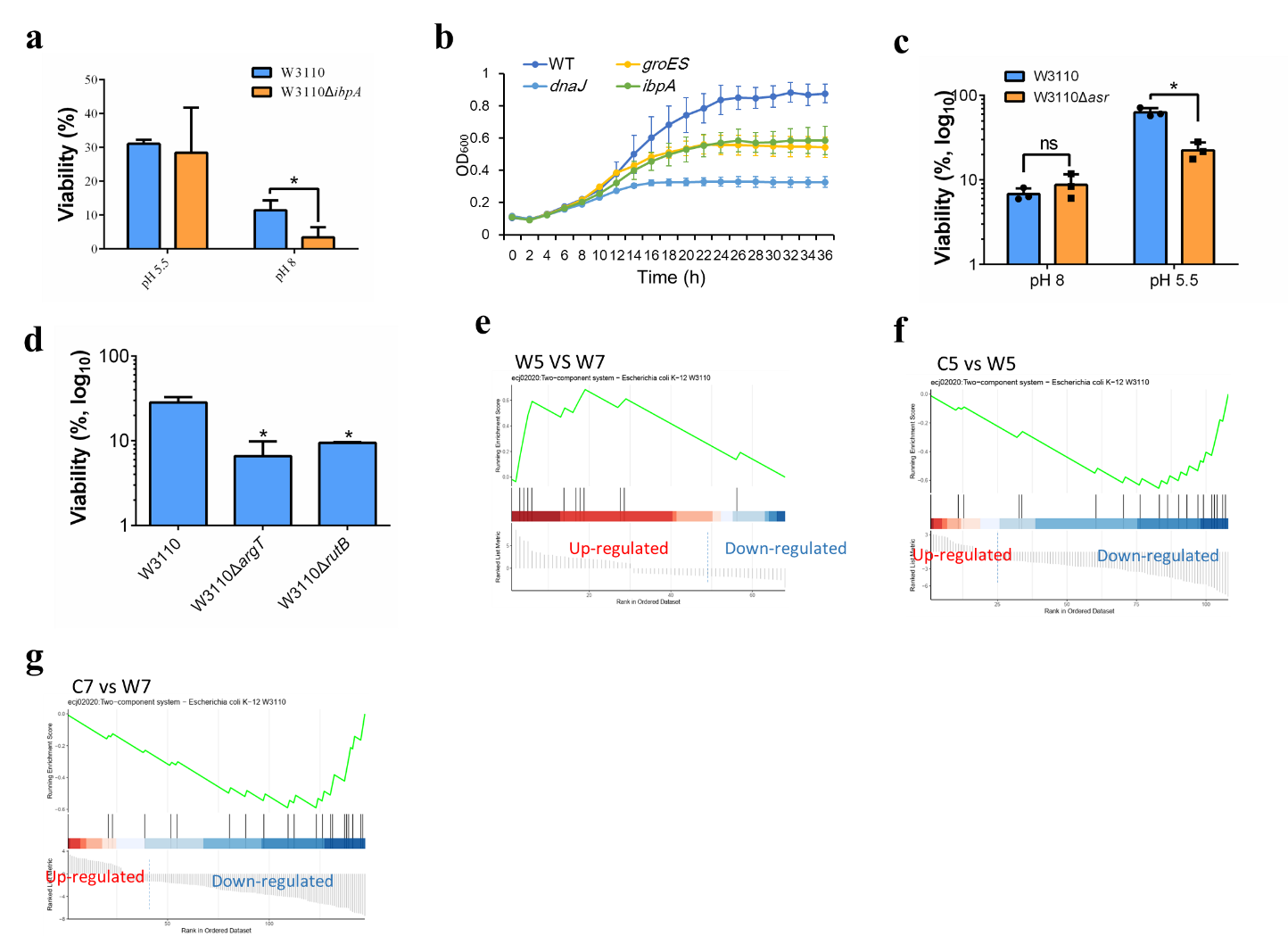


**Supplementary Figure 4. AR of membrane proteins. a** *ibpA* deletion decreases the acid resistance of stationary phase at pH 8. **b** Growth curves of W3110Δ*groES*, W3110Δ*dnaJ* and W3110Δ*ibpA* at pH 5.5 in EG media (n=4). **c** *asr* play a role in acid resistance of stationary phase at pH 8. **d** W3110Δ*argT* and W3110Δ*rutB* are acid resistance gene when grow to stationary phase at pH5.5. **e-g** Enrichment plots for the two-component systems genes related to different pH in W3110 and *crp*-deficient strains. Bars represent mean ± standard deviation (n = 3-4/group). ns, not significant; *, p< 0.05.
