# Supplementary material for: Quantitative transcriptomic and metabolic analyses reveal the roles of RpoS and Crp in the acid resistance system 1 in Escherichia coli
Source: Microbiol Spectr. 2026 Jun 15;14(7):e02063-25. doi: 10.1128/spectrum.02063-25 (PMC13339887; doi:10.1128/spectrum.02063-25)
Supplement: Supplemental tables — Tables S9 and S10. [file spectrum.02063-25-s0009.docx]

Supplementary Table 9 Bacterial strains and plasmids used in this study

| Strains or plasmids | Genotype or description | Source or reference |
| --- | --- | --- |
| Strains |  |  |
| W3110 | λ- F- derived from *E. coli* K-12 | Lab stock |
| BW25113 | lacIqrrnBT14 ΔlacZWJ16hsdR514  ΔaraBADAH33ΔrhaBADLD78 | (50) |
| BW25113/pKD46 |  | (50) |
| BW25113/pKD3 |  | (50) |
| BW25113/pKD4 |  | (50) |
| EK395 | K-12 *rpoS*::Tn10 | (2) |
| EF928 | K-12 Δ*cyaA*1400::Kan^r^ | (2) |
| EF528 | K-12 Δ*crp*::Cm^r^ | (2) |
| WB009 | W3110 *crp*∷Cm^r^ | This study |
| WB010 | W3110 *rpos*∷Cm^r^ | This study |
| WB011 | W3110 *wrbA*∷Cm^r^ | This study |
| WB012 | W3110 *yegP*∷Cm^r^ | This study |
| WB013 | W3110 *ibpA*∷Km^r^ | This study |
| WB014 | W3110 *ibpB*∷Cm^r^ | This study |
| WB015 | W3110 *dnaJ*∷Cm^r^ | This study |
| WB016 | W3110 *argT*∷Cm^r^ | This study |
| WB017 | W3110 *metK*∷Cm^r^ | This study |
| WB018 | W3110 *ycdM*∷Cm^r^ | This study |
| WB019 | W3110*yafN*∷Cm^r^ | This study |
| WB020 | W3110 *yeaR*∷Cm^r^ | This study |
| WB021 | W3110 *amtB*∷Cm^r^ | This study |
| WB022 | W3110 *metA*∷Cm^r^ | This study |
| WB023 | W3110 *yqgD*∷Cm^r^ | This study |
| WB024 | W3110 *ygiW*∷Cm^r^ | This study |
| WB025 | W3110 *metF*∷Cm^r^ | This study |
| WB026 | W3110 *wcaF*∷Cm^r^ | This study |
| WB027 | W3110 *yfbL*∷Cm^r^ | This study |
| WB028 | W3110 *narU*∷Cm^r^ | This study |
| WB029 | W3110 *asr*∷Cm^r^ | This study |
| WB030 | W3110 *ldcC*∷Cm^r^ | This study |
| WB031 | W3110 *emrK*∷Cm^r^ | This study |
| WB032 | W3110 *yhjX*∷Cm^r^ | This study |
| WB033 | W3110 *ycdL*∷Cm^r^ | This study |
| WB034 | W3110 *ydgD*∷Cm^r^ | This study |
| WB035 | W3110 *emrY*∷Cm^r^ | This study |
| WB036 | W3110 *yegZ*∷Cm^r^ | This study |
| WB037 | W3110 *groS*∷Cm^r^ | This study |
| WB038 | W3110 *ecpD*∷Cm^r^ | This study |
| WB039 | W3110 *aidB*∷Cm^r^ | This study |

1. Km^r^, resistant to kanamycin. Cm^r^, resistant to chloramphenicol.
2. Gift from Prof. Hiroshi Kobayashi

Supplementary Table 10 Primers used in this study

| Primer name | Sequence(5’→3’) |
| --- | --- |
| crp P1 | GAGACAGCGGCGTTATCTGGCTCTGGAGGAAAGCTTATA  ACAGAGGATAACCGCGCTGTGTAGGCTGGAGCTGCTTC |
| crp P2 | CACATCGGGGGAAACAAAATGGCGCGCTACCAGGTAAC  GCGCCACTCCGACGGGACATATGAATATCCTCCTTA |
| rpos P1 | GAACCAGGCTTTTGCTTGAATGTTCCGTCAAGGGATCAC  GGGTAGGAGCCACCTTTGTGTAGGCTGGAGCTGCTTC |
| rpos P2 | TGTGCACAGAAAAGGCCAGCCTCGCTTGAGACTGGCCTT  TCTGACAGATGCTTACCATATGAATATCCTCCTTA |
| wrbA P1 | TAAGTGGTAGCGAATCGCTACGGAATAGAGATAACACGAG  GAGTGGTTAGAAATGTGTGTAGGCTGGAGCTGCTTCG |
| wrbA P2 | CGACGTGATGAGCTTTCGCTTCTTGAGTTGGCATGCGTAT  CCTCCTGTTGAAGACATATGAATATCCTCCTTAG |
| yegP P1 | CAGCACCTTTAGATGCTGTTTTCGATACACAATTTCAATC  AAGGAGTCATTTGTGTAGGCTGGAGCTGCTTCG |
| yegP P2 | CTGTGGGGGGCTCTGGTAAAGCGCCGGACGCCAACAAC  CCACGCCCGGCATCGTAACATATGAATATCCTCCTTAG |
| ibpA p1 | CCTGTCCATTGTGGAAGGTCTTACATTCTCGCTGATTTCA  GGAGCTATTGATTTGTGTAGGCTGGAGCTGCTTCG |
| ibpA p2 | CATGGAGATGTCAGGCCGCGCCAGGCGGCCTTAGGGAA  TTAGTTGATTTCGATACCATATGAATATCCTCCTTAG |
| argT p1 | GCCATTTTCACTTGAGGGTTATGTATGAAGAAGTCGATTC  TCGCTCTGTCTTTGTGTGTAGGCTGGAGCTGCTTCG |
| argT p2 | CCTGGCGTGCACCATGATGATCATTCCATCAGGTACAGCT  TCCCAGCGACGTACATATGAATATCCTCCTTAG |
| dnaJ p1 | ATGGCTAAGCAAGATTATTACGAGATTTTAGGCGTTTCCAA  AACA GCGGAAGAGCTGTGTAGGCTGGAGCTGCTTCG |
| dnaJ p2 | TTAGCGGGTCAGGTCGTCAAAAAACTTCTTCACACCATCA  AAGAA GCTCTTTGAGCATATGAATATCCTCCTTAG |
| ibpB p1 | GCGAATCTTCGGATTTGCAGGTACTTACTCGCTTCTTAGA  AGGAGAAATGACTTGTGTAGGCTGGAGCTGCTTCG |
| ibpB p2 | CACCAGAATGGCGGGGCAAAGAGAATAGCTAGTTAGCTA  TTTAACGCGGGACATATGAATATCCTCCTTAG |
| metK p1 | ATGGCAAAACACCTTTTTACGTCCGAGTCCGTCTCTGAAG  GGCATCCTGACAAATGTGTAGGCTGGAGCTGCTTCG |
| metK p2 | GAGGCTGGCCTTTGAACGCAGGTGAAGAAAGATTACTTC  AGACGGCAGCATCCATATGAATATCCTCCTTAG |
| ycdM p1 | ATGCAGGATGCAGCGCCCCGTCTGACTTTCACTTTACGAG  ACGAAGAGAGGTTGTGTGTAGGCTGGAGCTGCTTCG |
| ycdM p2 | CACTTTGCTGCGGATCGAAGGTAATGGCTTCCGGTCGAG  CGGTTAAGGTCGTCACATATGAATATCCTCCTTAG |
| yafN p1 | GATGTATACTATTATGTATATTCTGGTGTGCATTATTATGA  GGGTATCACTGTTGTGTAGGCTGGAGCTGCTTCG |
| yafN p2 | GTTCCTCTGCTGTAAGTTGCAGGCGAATAAGTTTTGTT  TTGAATACCCGCATCCCATATGAATATCCTCCTTAG |
| yeaR p1 | CAGCGCATTCACCGAAGGAGGGAAAAGGATGCTTCAA  ATCCCACAGAATTATATGTGTAGGCTGGAGCTGCTTCG |
| yeaR p2 | CGCCATTGCTGTTATTGGTGACGGTCACGGTATACGTT  GCTTTGCCCATGATCATATGAATATCCTCCTTAG |
| amtB p1 | CTGGCGATGCTTCCGGGACTGGTAATGGCTGCACCTGCGG  TGGCCGATAAAGCCGACTGTGTAGGCTGGAGCTGCTTCG |
| amtB p2 | GCTGTTGACATCCAGCCCTTCTCGCTCCTGCTCTTCCGGTA  CACGCAGACAGACCAACCGCATATGAATATCCTCCTTAG |
| metA p1 | CTTCAGCTATCTGGATGTCTAAACGTATAAGCGTATGTAGT  GAGGTAATCAGGTTTGTGTAGGCTGGAGCTGCTTCG |
| metA p2 | CGAAGGTGCCTGAGGTAAGGTGCTGAATCGCTTAACGAT  CGACTATCACAGAAGACATATGAATATCCTCCTTAG |
| yqgD p1 | GAGAATTTGGTTAGCTCAAACTGTTGTGTGGATTTTCT  GTGGTAGCGGATCCTGTGTAGGCTGGAGCTGCTTCG |
| yqgD p2 | CCCGGCTGGAAGTGGCAACACGAAAGAAACGTCGTG  TGCTTTTTATTTAAGCCATATGAATATCCTCCTTAG |
| metF p1 | GGATAGATGTGCACAACACAACATATAACTACAAGCG  ATTGATGAGGTAAGGTTGTGTAGGCTGGAGCTGCTTCG |
| metF p2 | GCCACACTATTTATAAACCAGGTCGAACCCCCAGCGTA  TGGCAAATCGCGTAACCATATGAATATCCTCCTTAG |
| wcaF p1 | CTACCTCAAAGACGAAAGCCTTATATAACAAAGTCTGA  ATATAAGGAAAACCATGTGTAGGCTGGAGCTGCTTCG |
| wcaF p2 | CACCGGTGATGAGAGCGACTTTTGACATGTATTATTCCT  CTGTATTTTTGAATCATATGAATATCCTCCTTAG |
| yfbL p1 | CGAAACCCATAGCAAATAATGATTCAACAACGCAACCC  ATTAATAATTTGCATTGTGTAGGCTGGAGCTGCTTCG |
| yfbL p2 | TTATTTACTGTTGTATAACAAAGTTATAACACCATCCACT  ACCTGAGCCATTCATATGAATATCCTCCTTAG |
| narU p1 | CATTACCAATGTGTGCATGTGAGGAACAATATGGCACTG  CAAAATGAGAAATGTGTAGGCTGGAGCTGCTTCG |
| narU p2 | GATCTGATGCCTTGTCGGATGCGACACGGTTGTACATCA  GGCATCGATCTCTTACATATGAATATCCTCCTTAG |
| asr p1 | GAGGGTATGACAATGAAAAAAGTATTAGCTCTGGTTGT  TGCCGCTGCTATGTGTGTAGGCTGGAGCTGCTTCG |
| asr p2 | TTACGCTGCGGGTTGTGCAGCAGGTTTTGCCGGTTGCT  GATGGCTGTGTTTCCATATGAATATCCTCCTTAG |
| ldcC p1 | ATGAACATCATTGCCATTATGGGACCGCATGGCGTCTTT  TATAAAGATGAGCCCATGTGTAGGCTGGAGCTGCTTCG |
| ldcC p2 | TTATCCCGCCATTTTTAGGACTCGTACGCGGTAAACGCC  GTCTTCGTCCTGTTTCCATATGAATATCCTCCTTAG |
| ygiW p1 | GTTAAGGACTATCTTGTTAAATGCTCGGAAACAGACATT  AAAGGGAGTAATAAACTGTGTAGGCTGGAGCTGCTTCG |
| ygiW p2 | TTACGGATTTACTTTGCGGATCTGTTTGACGTCAATTTC  AACAGAATTCCAGCATATGAATATCCTCCTTAG |
| emrK p1 | CATCTATGAGAAATGAGAGATAATAGTGGAACAGATTA  ATTCAAATAAAATGTGTAGGCTGGAGCTGCTTCG |
| emrK p2 | CACCATAACGTCCCACCGGTTAATGGTGCCGGAGTTG  ATTTAGTGATTGCCACATATGAATATCCTCCTTAG |
| yhjX p1 | CAGGAATACTGCCATGACACCTTCAAATTATCAGCGTA  CCCGCTGGCTGACACTGTGTAGGCTGGAGCTGCTTCG |
| yhjX p2 | TTAAAGGGAGCCATGCGCCTCACGCAACATTTTCTGCT  CTGGCTGACGAATCCATATGAATATCCTCCTTAG |
| ycdL p1 | GCCCATCTACCTGCGCTGACTCAGGAGGTGGCATGATG  ACGACCTTAACCTGTGTAGGCTGGAGCTGCTTCG |
| ycdL p2 | AACTCCTTAAGCGATATGAGCAAAGGACGTGGGAGAA  AGCGCGTCGCAGAATCATATGAATATCCTCCTTAG |
| ydgD p1 | AATCATAATTACCCACCAGAGTGTGATATGCGTACAAC  CATTGCTGTAGTGTGTGTAGGCTGGAGCTGCTTCG |
| ydgD p2 | CGCTGTCGGGCAGCGTTTGAACATTATTTTTGCGACAG  TTGATCCAGCTTGCATATGAATATCCTCCTTAG |
| emrY p1 | ATGGCAATCACTAAATCAACTCCGGCACCATTAACCGG  TGGGACGTTATGGTGTGTGTAGGCTGGAGCTGCTTCG |
| emrY p2 | CTCCTTTTATCATCACCCAACGCCTTTCGCTGTAAACG  GCGGTTTCGCAAACATATGAATATCCTCCTTAG |
| yegZ p1 | CTATCGATGTGGTCAATGGAAGACGGTTACCAGAGAT  AGGGCTTATGCATAATGTGTAGGCTGGAGCTGCTTCG |
| yegZ p2 | TACCCTTACAAAACAAACAGATAAAATAAAAACATC  ACTCATTATCTTCTGCATATGAATATCCTCCTTAG |
| groS p1 | CTCCGGCGTCACCCATAACAGATACGGACTTTCTCA  AAGGAGAGTTATCATGTGTAGGCTGGAGCTGCTTCG |
| groS p2 | GCTGCCATTATCTTTATTCCTTAAATTCGTATGTTCAG  TGTCGTGCGCGGACATATGAATATCCTCCTTAG |
| ecpD p1 | GACGCAGACAGGAGAAGAGAATGAGAGTTAACCTACT  AATAACGATGATAATGTGTAGGCTGGAGCTGCTTCG |
| ecpD p2 | TTAGTTAATGTTACGCCACGTCGCCTGAACATGAATCT  CTCCTG AAGCGCTGACCATATGAATATCCTCCTTAG |
| aidB p1 | GTGCACTGGCAAACTCACACCGTTTTTAATCAACCTAT  ACCAT TAAATAACAGTGTGTAGGCTGGAGCTGCTTCG |
| aidB p2 | TTACACACACACTCCCCCCGTCGCCCGCAGCAATAAA  TCATTCT GGATCTGCTCCATATGAATATCCTCCTTAG |
| Cm inbox | TCTTGCCCGCCTGATGAATGCTC |
